# Analysis of the Tumor Immune Microenvironment (TIME) in Clear Cell Renal Cell Carcinoma (ccRCC) Reveals an M0 Macrophage-Enriched Subtype: An Exploration of Prognostic and Biological Characteristics of This Immune Phenotype

**DOI:** 10.3390/cancers15235530

**Published:** 2023-11-22

**Authors:** Mark Farha, Srinivas Nallandhighal, Randy Vince, Brittney Cotta, Judith Stangl-Kremser, Daniel Triner, Todd M. Morgan, Ganesh S. Palapattu, Marcin Cieslik, Ulka Vaishampayan, Aaron M. Udager, Simpa S. Salami

**Affiliations:** 1 Department of Medical Education, University of Michigan Medical School, Ann Arbor, MI 48109, USA; vvu9002@nyp.org (M.F.); vaishamu@med.umich.edu (U.V.); 2 Department of Urology, Michigan Medicine, Ann Arbor, MI 48109, USA; 3 Department of Urology, Medical University of Vienna, 1090 Vienna, Austria; 4 Rogel Cancer Center, University of Michigan, Ann Arbor, MI 48109, USA; mcieslik@med.umich.edu (M.C.); udager@med.umich.edu (A.M.U.); 5 Department of Pathology, Michigan Medicine, Ann Arbor, MI 48109, USA; 6 Michigan Center for Translational Pathology, Michigan Medicine, Ann Arbor, MI 48109, USA; 7 Department of Medicine, Michigan Medicine, Ann Arbor, MI 48109, USA

**Keywords:** clear cell renal cell carcinoma (ccRCC), tumor immune microenvironment (TIME), macrophages, genomics, immune checkpoint blockade, biomarkers

## Abstract

**Simple Summary:**

Until recently, checkpoint inhibitors have largely been reserved for the metastatic disease space in clear cell renal cell carcinoma (ccRCC). Over the last several years, a growing body of trials have investigated the application of checkpoint inhibitor therapy in the neoadjuvant and adjuvant settings. However, these trials have yielded mixed results, due largely in part to a lack of high-fidelity biomarkers for patient selection and response prediction. Here, we present a simple workflow leveraging genomic data to characterize the immune microenvironment in ccRCC and identify a key macrophage subset that is associated with aggressive tumor biology, poor prognosis, and decreased response to immune checkpoint blockade (ICB). This work lays the foundation for future mechanistic discovery and prospective validation of integrating macrophage content into a ccRCC immune checkpoint blockade biomarker strategy.

**Abstract:**

There is a need to optimize the treatment of clear cell renal cell carcinoma (ccRCC) patients at high recurrence risk after nephrectomy. We sought to elucidate the tumor immune microenvironment (TIME) of localized ccRCC and understand the prognostic and predictive characteristics of certain features. The discovery cohort was clinically localized patients in the TCGA-Kidney Renal Clear Cell Carcinoma (KIRC) project (*n* = 382). We identified an M0 macrophage-enriched cluster (*n* = 25) in the TCGA-KIRC cohort. This cluster’s median progression-free survival (PFS) and overall survival (OS) were 40.4 and 45.3 months, respectively, but this was not reached in the others (*p* = 0.0003 and <0.0001, respectively). Gene set enrichment (GSEA) analysis revealed an enrichment of epithelial to mesenchymal transition and cell cycle progression genes within this cluster, and these patients also had a lower predicted response to immune checkpoint blockade (ICB) (4% vs. 20–34%). An M0-enriched cluster (*n* = 9) with shorter PFS (*p* = 0.0006) was also identified in the Clinical Proteomics Tumor Analysis Consortium (CPTAC) cohort (*n* = 94). Through this characterization of the TIME in ccRCC, a cluster of patients defined by enrichment in M0 macrophages was identified that demonstrated poor prognosis and lower predicted ICB response. Pending further validation, this signature can identify localized ccRCC patients at high risk of recurrence after nephrectomy and who may require therapeutic approaches beyond ICB monotherapy.

## 1. Introduction

Cancer of the kidney and renal pelvis is one of the most common cancers diagnosed in the United States [1], of which renal cell carcinoma (RCC) accounts for 90% [2]. The clear cell RCC (ccRCC) subtype accounts for ~75% of RCCs [3]. While the number of patients presenting with metastatic disease is decreasing due in large part to more liberal use of abdominal imaging [4], recurrence after nephrectomy is a relatively common incidence, with it occurring in about 20% of patients with localized disease at diagnosis [4] and 35–47% with pathologic T2–T4 disease [5]. Patients with similar pathologic stage at nephrectomy can have vastly different outcomes with respect to recurrence [6]. At the time of diagnosis, the tumor (T) stage is an independent prognostic factor, with a 7% incidence of recurrence in T1 compared to 39% in T3 tumors [7]. With such a high rate of relapse after nephrectomy, there is a clear unmet need to optimize adjuvant therapy for these patients to minimize recurrence risk.

The adjuvant treatment paradigm has undergone a massive revolution in recent years, especially with the advent of immunotherapy. Prior systemic treatment for ccRCC has included recombinant IL-2 and interferon, anti-angiogenic agents such as sunitinib, and mTOR inhibitors such as everolimus. Numerous trials have been undertaken for anti-VEGF therapies in the adjuvant setting, including ASSURE, PROTECT, ATLAS, SORCE, and S-TRAC. Clear cell RCC, however, is one of the most heavily immune infiltrated cancer types, making it an extremely appealing malignancy to study immune-modulatory therapeutic approaches [8]. Immune checkpoint blockade (ICB) agents targeting the PD-1/PD-L1 axis or CTLA-4 have been extensively studied in the metastatic setting, but their introduction to high risk localized RCC has been more recent. After the positive interim analysis of the KEYNOTE 564 trial [9], the FDA approved pembrolizumab for the adjuvant treatment of localized RCC at high risk of recurrence in late 2021. Several trials of adjuvant ICB in RCC are ongoing, including PROSPER RCC, RAMPART, CheckMate-914, and IMmotion010. However, the failure of Atezolizumab to show benefit over placebo in the IMmotion 010 trial [10] despite encouraging results from KEYNOTE 564, reinforces the critical need for biomarkers to identify patients most suitable for immunotherapy. 

Although applications of ICB to RCC are expanding, response prediction remains challenging. Biomarkers traditionally used for ICB response prediction are not as readily applicable to RCC. With respect to PD-1/PD-L1 expression, there are different quantification methods, and the positivity rate ranges vastly in various trials. RCC has a lower tumor mutational burden than other solid tumors and the incidence of microsatellite instability (MSI) is <2% in ccRCC [11]. While some efforts are ongoing to utilize gene signatures to predict response to ICB therapy [12], an understanding of the TIME and how it relates to recurrence risk and ICB response is essential given that RCC has one of the highest degrees of immune infiltration of any cancer. A signature based on the tumor immune microenvironment (TIME) presents an exciting opportunity to close the gap and improve the projection of recurrence risk and patient selection for adjuvant therapy in those with high-risk, localized ccRCC.

The wide availability of transcriptomic data and the development of gene expression-based immune cell deconvolution methods, such as CIBERSORT [13], provide a unique opportunity to comprehensively characterize the (TIME) in ccRCC. Here, publicly available RNA sequencing (RNASeq) data from the Cancer Genome Atlas (TCGA) were used to impute immune cell subsets within the ccRCC cohort using CIBERSORT. Unsupervised hierarchical clustering was then carried out on the individual samples and a macrophage-enriched cluster was identified and found to be prognostic of poor survival in both the discovery and a separate validation cohort (the Clinical Proteomic Tumor Analysis Consortium, CPTAC). We then further characterized this cluster using gene set enrichment analysis (GSEA) and utilized an ancillary gene signature-based tool, Tumor Immune Dysfunction and Exclusion (TIDE), to predict potential response to ICB therapy.

## 2. Materials and Methods

### 2.1. Discovery Dataset

We analyzed patients with clinically localized ccRCC in the TCGA–Kidney Renal Clear Cell Carcinoma (KIRC) project cohort with available mRNA expression data. A subset of TCGA-KIRC patients have since been identified on further pathology re-review as suspicious for non-clear cell histology. These samples were thus excluded from the study (discovery dataset *n* = 382) [14]. All analyses were performed using R software for statistical computing (R Version 3.6.3, Vienna, Austria).

The TCGA-KIRC dataset contains clinical, pathologic, and genomic data. A mixture file containing RNASeq analysis by Expectation-Maximization (RSEM) gene expression data from the samples in TCGA-KIRC was downloaded from cBioPortal [15]. The TCGAbiolinks package was used to retrieve clinical data from the National Cancer Institute Genomic Data Commons repository [16,17,18]. To remove irrelevant or noisy genes in our expression analysis, we utilized a counts per million (CPM) > 1 filter to remove genes with low expression. We also used a library size cut-off; samples with a library size below 1 million reads were removed.

### 2.2. Clustering Based on Immune Cell Subpopulations

Immune cell deconvolution was performed on the discovery dataset using the CIBERSORT in silico flow cytometry tool [13]. CIBERSORT is an online resource that utilizes a gene expression signature matrix, consisting of expression profiles of 547 genes, termed the LM22, to “deconvolute” or estimate the relative fractions of 22 unique immune cell types and activation states in complex mixtures from bulk RNA-seq data [19]. CIBERSORT implements a machine learning approach and has been found to be more accurate in benchmarking studies than other methods in resolution for closely related cell types and complex mixtures with a high degree of noise (such as tumors) [13]. CIBERSORT has been validated against traditional flow cytometric and immunohistochemical methodologies and was found to detect immune composition of queried sample with a high degree of accuracy, both in idealized samples and admixtures with varying degrees of noise to simulate tumors [13]. It has since been utilized in many landmark scientific papers since its creation without additional validation steps in a basic science laboratory, for example, as a part of biomarker analysis from Checkmate trials in ccRCC [20]. Additionally, CIBERSORT has been applied to a pan-cancer cohort (PRECOG—PREdiction of Clinical Outcomes from Genomic Profiles) of 6000 specimens, including 300 RCC patients, demonstrating a precedent for application of CIBERSORT in RCC [21]. The number of permutations for the deconvolution was set to 100 as described in prior CIBERSORT vignettes. The R package ComplexHeatmap [22] was used to perform unsupervised hierarchical clustering on the individual samples in the dataset; the clustering distance metric was set to the maximum distance between rows, and the clustering method chosen was Ward’s minimum variance. The relative fraction of each of the immune cells was used to cluster samples with similar immune content, and an M0-enriched cluster emerged. Within the 547 genes of LM22, genes that were significantly differentially expressed in M0 macrophages include: ACP5, BHLHE41, C5AR1, CCDC102B, CCL22, CCL7, COL8A2, CSF1, CXCL3, CXCL5, CYP27A1, DCSTAMP, GPC4, HK3, IGSF6, MARCO, MMP9, NCF2, PLA2G7, PPBP, QPCT, SLAMF8, SLC12A8, TNFSF14, and VNN1 [19] (Supplementary Materials, Figure S1). The optimal number of clusters was determined based on scree plot analysis. Scree plots show within-cluster variability on the *y*-axis and the number of clusters on the *x*-axis. The optimal number of clusters is at the “elbow” of the curve, where the within-cluster variability is at a minimum, and increasing the number of clusters does not considerably reduce within-cluster variability. The scree plot “elbow method” was used to determine the optimal number of clusters for both the CPTAC and TCGA cohorts. 

### 2.3. Survival Analysis by Clusters

The primary outcome was progression-free survival (PFS), defined as the time from pathologic diagnosis to new tumor event, which includes disease progression, locoregional recurrence, distant metastasis, new primary tumor, or death from tumor [23]. Overall survival (OS) was also included as an endpoint, defined as the time from pathologic diagnosis to death from any cause [23]. Patients still alive were censored at the time of last follow-up. Each endpoint was assessed using the Kaplan–Meier method by R package survival [24], and survival curves were compared using the Mantel–Cox log-rank test.

### 2.4. Validation Dataset

The unsupervised hierarchical clustering and subsequent survival analysis of the resultant clusters were validated in a cohort of patients with localized, treatment-naïve ccRCC from the Clinical Proteomics Tumor Analysis Consortium (CPTAC) group (validation dataset, *n* = 99) [25]. Publicly available clinical and transcriptomic data were downloaded and analyzed as described for the discovery dataset. To ensure comparability of the discovery and validation datasets, we utilized the removeBatcheffect function of the R limma package to adjust for cohort-specific batch effects between TCGA and CPTAC.

### 2.5. Tumor Immune Characterization Using TIDE

The TIDE tool was used to characterize the TIME further and predict ICB treatment response in the TCGA-KIRC cohort. TIDE uses a gene expression signature developed using immunotherapy-treated melanoma patients to predict response to ICB therapy [26]. TIDE performance has been validated on non-small-cell lung cancer datasets in addition to melanoma and can be extrapolated to other cancer types based on the underlying assumptions of the model. Z-score transformed RSEM gene expression with z-score transformation was used as TIDE input. Output includes the prediction of immunotherapy response and scores correlating with the expression of gene signatures associated with immune dysfunction.

### 2.6. Gene Set Enrichment Analysis (GSEA)

Differentially expressed gene (DEG) analysis was performed using the Shiny web application GENAVI [27]. Raw counts and metadata files were created, and DEG analysis was performed using the DESeq2 method, with non-cluster 4 as the reference. Log_2_fold change values from the DEG analysis were used to perform gene set enrichment analysis (GSEA) with MSigDb pathways, specifically the H: hallmark gene sets collection [28]. The Benjaminin–Hochberg (BH) test was used for multiple corrections and a false discovery rate adjusted *p*-value cut-off of 5% was used to determine statistical significance. The R package ggplot2 package [29] was then used to visualize the enrichment scores of significantly overrepresented or underrepresented gene sets.

### 2.7. Multivariable Cox Hazards Analyses 

A multivariable cox hazards analysis adjusting for clinicopathologic variables such as age, sex, grade, and stage was performed using R package survival. The *p*-value cut-off was set at 0.05, and the data were visually represented using R package (version 0.4.9) survminer.

## 3. Results

### 3.1. Cohort Description

The demographic, pathologic, and molecular characteristics of the discovery cohort (TCGA-KIRC) are outlined in Table 1. The mean age of the patients included in the cohort at the time of diagnosis was 60.8 years, and 64% were male. The racial mix was heavily skewed towards White patients (95%). There was a predominance of earlier stage disease (Stage I—54% and Stage II—12%) and an equal mix of high and low grades (G1—3%, G2—45%/G3—37%, and G4—10%). Key molecular alterations observed in the cohort were mutations in *p53* (15%), *VHL* (61%), *PBRM1* (35%), *SETD2* (16%), and *TCEB1* (1%). A description of the CPTAC cohort is included in Table 2.

### 3.2. Hierarchical Clustering Based on the Immune Cell Subsets Identified Macrophage-Enriched Clusters

**Figure 1:**
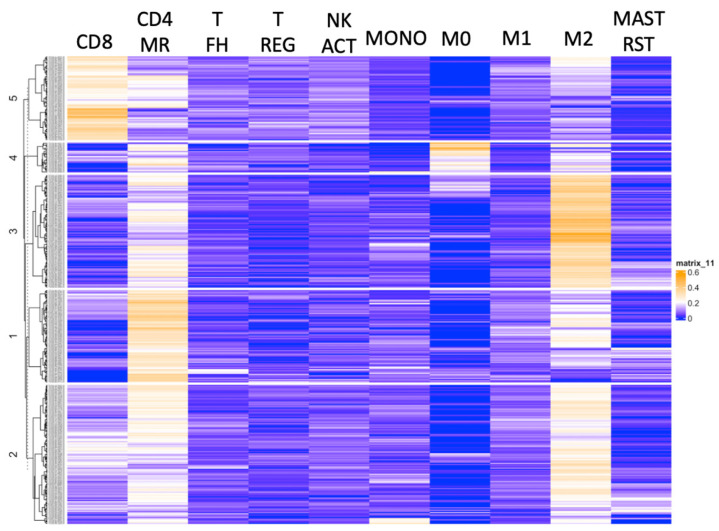
Heatmap displaying the relative abundance of the ten most prevalent immune cell subsets in non-metastatic ccRCC patients in the TCGA-KIRC dataset. The columns display the relative fraction of each individual immune cell, while the rows display unique identifiers within each cluster. Abbreviations: CD4: CD4 memory resting cells, T FH: T follicular helper cells, T REG: regulatory T cells, NK ACT: active NK cells, MONO: monocytes, M0–M2: macrophage subsets, and MAST RST: resting mast cells.

The patients were clustered based on relative fractions of immune cell subsets: cluster 1 (M2 macrophage^Mid^, CD4-MR^Hi^; *n* = 77), cluster 2 (M2 macrophage^Mid^, CD4-MR^Mid^; *n* = 116), cluster 3 (M2 macrophage^Hi^; *n* = 94), cluster 4 (M0 macrophage^Hi^, M2 macrophage^Mid^; *n* = 25), and cluster 5 (CD8^Hi^; *n* = 70) as shown in Figure 1. The clustering diagram for the CPTAC validation cohort is shown in the supplement (Supplementary Materials, Figure S2). Notably, a cluster enriched in M0 and M2 macrophages (*n* = 9) was identified in this independent cohort as well. 

### 3.3. M0 Macrophage Enrichment Is Associated with Shorter PFS in Two Separate Cohorts

**Figure 2:**
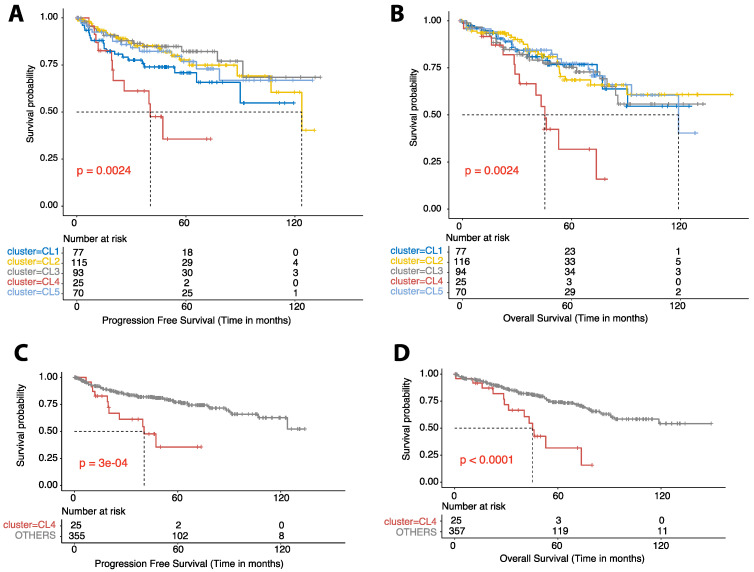
Survival analyses. Kaplan–Meier curves for progression-free survival (PFS) (**A**) and overall survival (OS) (**B**) for each cluster in the TCGA-KIRC cohort are shown. Pairwise log-rank comparisons were conducted for each curve. Kaplan–Meier curves for (PFS) (**C**) and (OS) (**D**) for grouped clusters 1, 2, 3, and 5 combined and labeled as OTHER compared with cluster 4 are shown. Pairwise log-rank comparisons are included for each curve.

**Figure 3:**
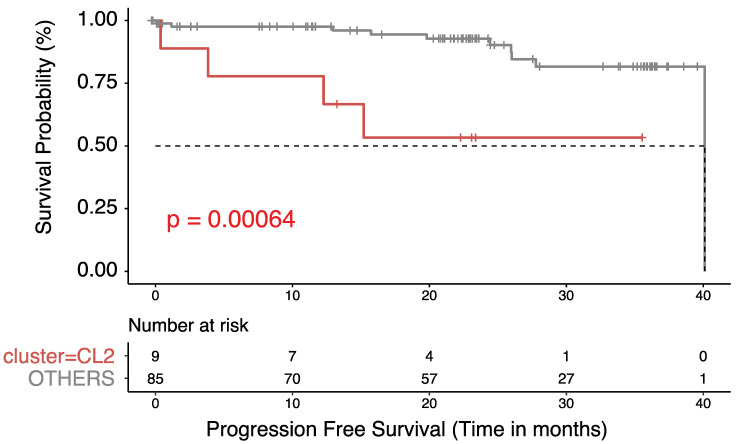
Kaplan–Meier curves for (PFS) for grouped clusters 1 and 3–6 combined and labeled as OTHER compared with cluster 2^M0-Hi^ in the CPTAC cohort are shown. A pairwise log-rank test was used for comparison.

**Figure 4:**
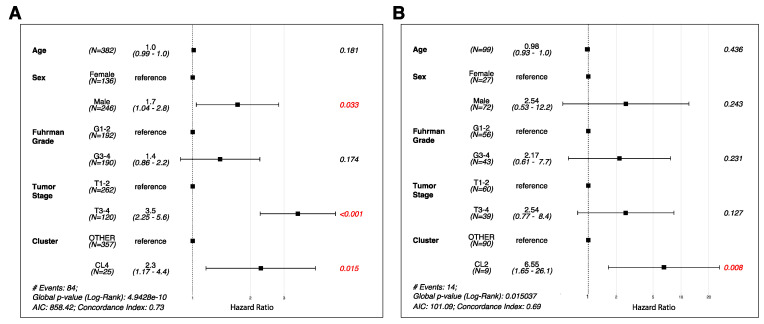
(**A**,**B**). Multivariable analysis with the hazard ratio (HR) represented in a forest plot adjusting for age, pathologic T-stage, grade, and sex are shown for the (**A**) TCGA and (**B**) CPTAC cohorts. TIME clusters and clinicopathologic variables are located on the *y*-axis while hazard ratios are located on the *x*-axis. Red text indicates significant covariates in the analysis.

The median follow-up time for all patients was 45.5 months and was similar across the clusters (46, 45.7, 45.5, 46.3, and 46.1 for clusters 1–5, respectively). Overall, 84 patients had a progression event (22%), and 99 patients (26%) had died at the time of this analysis. The median time between pathologic diagnosis and progression was not reached (NR), 123.8, NR, 40.4, and NR months in clusters 1–5, respectively (*p* = 0.0024; Figure 2A). When the clusters were grouped, the median PFS was 40.4 months in cluster 4^M0-Hi^ and NR in the other clusters (*p* = 0.0003; Figure 2C). Pairwise comparisons via log-rank tests revealed that time to progression in cluster 4 ^M0-Hi^ was significantly shorter than all other clusters except for cluster 1^M2-Mid,CD4-MR-Hi^ (cluster 5^CD8-Hi^ vs. 4^M0-Hi^, 0.0089; cluster 3^M2-Hi^ vs. 4^M0-Hi^, 0.0016; cluster 2^M2-Mid,CD4-MR-Mid^ vs. 4^M0-Hi^, 0.0016; cluster 1^M2-Mid,CD4-MR-Hi^ vs. 4^M0-Hi^–0.1123; *p*-value adjustment method BH). The median time between pathologic diagnosis and death was NR, NR, NR, 45.3, and 118.8 months in clusters 1–5, respectively (*p* = 0.0024; Figure 2B). Pairwise comparisons using log-tank tests revealed that time to death in cluster 4 was significantly shorter than in all other clusters (cluster 5^CD8-Hi^ vs. 4^M0-Hi^, 0.0019; cluster 3^M2-Hi^ vs. 4^M0-Hi^, 0.0037; cluster 2^M2-Mid,CD4-MR-Mid^ vs. 4^M0-Hi^, 0.0019; cluster 1^M2-Mid,CD4-MR-Hi^ vs. 4^M0-Hi^, 0.0029; *p*-value adjustment method BH). Furthermore, the median OS was 45.3 months in cluster 4^M0-Hi^ and NR in the other clusters combined (i.e., clusters 1–3 and 5 were combined given the similar clinical survival curves (*p* ≤ 0.0001; Figure 2D). Similarly, in the CPTAC cohort, cluster 2^M0-Hi^ had a significantly shorter PFS than all other clusters combined (i.e., clusters 1, 3–6) (*p* = 0.00064; Figure 3). After establishing that M0 macrophage-enriched ccRCC is associated with the poorest prognosis of all immune subtypes, a multivariable analysis was performed to assess the independent prognostic impact of the TIME clusters on PFS. This was achieved in both CPTAC and TCGA cohorts (Figure 4A,B). In TCGA, the M0 macrophage-enriched cluster was independently associated with PFS (using the other clusters as a reference, HR 2.3, 95% CI 1.17–4.40, *p* = 0.015). Similar results were replicated in CPTAC (using the other clusters as a reference, HR 6.55, 95% CI 1.65–26.1, *p* = 0.008).

### 3.4. M0 Macrophage Enrichment Is Also Associated with Other Pro-Tumorigenic TME Characteristics 

**Figure 5:**
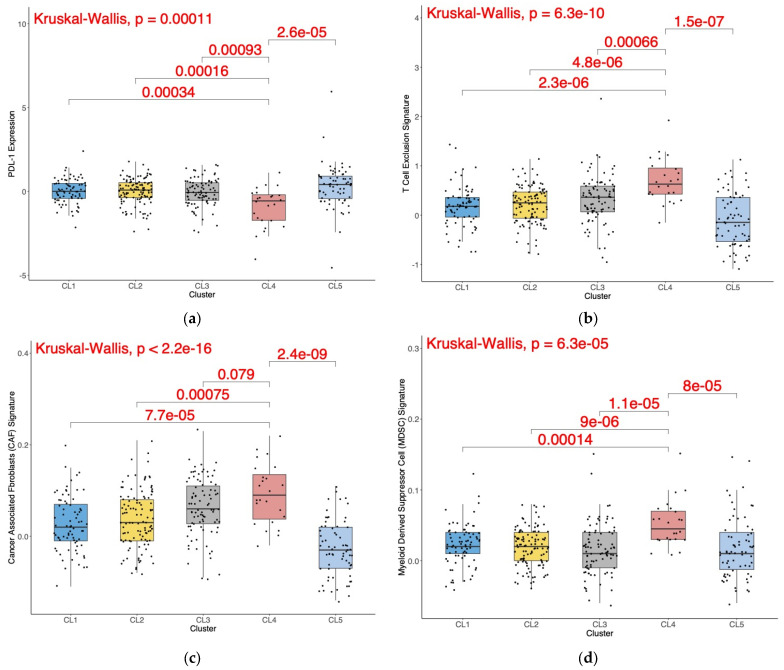
(**a**–**d**) Box plots representing key tumor immune microenvironment parameters by cluster in the non-metastatic TCGA-KIRC cohort. Data presented in this figure are from the tumor immune dysfunction and exclusion (TIDE) module. PD-L1 expression Z-scores (**a**) and signatures for T-cell exclusion (**b**) and the prevalence of cancer-associated fibroblasts (CAFs) (**c**) and myeloid-derived suppressor cells (MDSCs) (**d**) are included. The global Kruskal–Wallis *p*-value as well as individual pairwise Wilcoxon test *p*-values for pairwise tests between cluster 4 and the other individual clusters are displayed.

The TIDE tool was then utilized to analyze the RNA-Seq data to assess transcriptomic biomarkers related to response to ICB therapy. As shown in Figure 5, subjects in cluster 4^M0-Hi^ had significantly lower tumoral PD-L1 expression (Kruskal–Wallis *p* = 0.00011; Figure 5a), a significantly higher T-cell exclusion signature (Kruskal–Wallis *p* = 6.3 × 10^−10^; Figure 5b), and significantly higher cancer-associated fibroblast (CAF) transcriptomic signatures (Kruskal–Wallis *p* = 2.2 × 10^−16^; Figure 5c) and myeloid-derived suppressor cells (MDSCs) (Kruskal–Wallis *p* = 6.3 × 10^−5^; Figure 5d) in the stroma. Although the TCGA-KIRC cohort was ICB-naïve, cluster 4^M0-Hi^ had the lowest predicted response rate to ICB (4%, Table 1).

**Figure 6:**
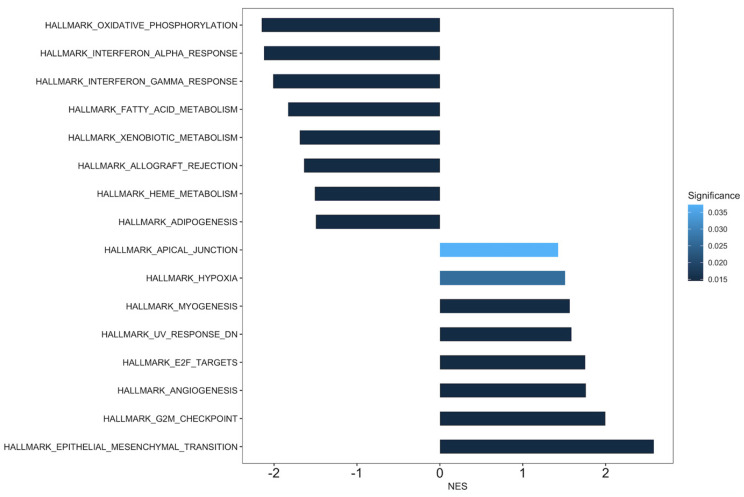
Results of gene set enrichment analysis (GSEA) for MsigDB hallmark gene sets comparing cluster 4 to clusters 1, 2, 3, and 5 combined in the non-metastatic TCGA cohort. The normalized enrichment scores (NESs) are represented on the *x*-axis. Positive and negative NESs indicate enriched and suppressed gene sets, respectively, with rightward facing bars indicating enriched gene sets and leftwards facing bars indicating suppressed gene sets. The bars are shaded by the FDR-adjusted *p*-value. Only significant gene sets are presented.

GSEA was performed, with it comparing cluster 4^M0-Hi,M2-Mid^ with the other clusters combined as the reference. Significantly over-enriched and under-enriched hallmark gene sets are presented in Figure 6. Notably, the over-enriched gene sets in cluster 4^M0-Hi,M2-Mid^ were pro-tumorigenic pathways, including cell cycle progression, angiogenesis, and epithelial to mesenchymal transition. Under-enriched gene sets include those related to metabolic pathways and interferon response.

## 4. Discussion

In this study, we identified an M0 macrophage-enriched ccRCC cluster that is prognostic of poor survival in two separate cohorts. This cluster demonstrated enrichment of pro-tumorigenic transcriptomic biomarkers and gene sets associated with cancer progression, including angiogenesis, epithelial to mesenchymal transition, and cell cycle progression. Tumor-associated macrophages (TAMs) are regarded as orchestrators of crucial events necessary for cancer progression. These events include skewing adaptive responses, cell growth, angiogenesis, and extracellular matrix remodeling—changes that lead to establishing the premetastatic niche [30,31]. At the most basic level, macrophages are separated into an M1 subtype, which is proinflammatory and antifibrotic, and the M2 subtype, which is anti-inflammatory and profibrotic. Emerging evidence suggests that this classification scheme is an overly simplistic view of the role of TAMs in the TIME, and it is now clear that TAMs exist on a continuum of phenotypes with differing expressions of various activation markers and co-stimulatory receptors. Comprehensive classification of TAMs in ccRCC was recently reported by Chevrier et al. [32]. Correlation analysis of 17 TAM phenotypes identified revealed an association between pro-tumor macrophage phenotypes and decreased PFS [32]. However, the mass cytometry approach is difficult to reproduce at a large scale, and the clinical implications of their findings have not been validated [32]. With the ubiquitous nature of RNA-seq data and gene expression-based immune cell deconvolution methods like CIBERSORT, we capitalized on the unique opportunity to define immune phenotypes associated with poor outcomes and tested their clinical implications in the management of patients with ccRCC.

The distinguishing feature of the immune cluster described in our work is the M0 macrophage. While M0 macrophages are classically viewed as resting, non-polarized macrophages which require stimulation to take on the M1 or M2 phenotype [33], here, we suggest that M0 macrophages play a more active role as an early player central to establishing a hostile pro-tumor/anti-CD8 effector cell microenvironment. Multiple studies have demonstrated that cancer-associated fibroblasts (CAFs) promote macrophage polarization to the M2-like phenotype. We hypothesize that M0 macrophages act as early M2 macrophages; yet, they are still their own distinct entity with differences in chemokine expression [34]. This is supported by the prior literature in other cancer types [35,36] suggesting that M0 macrophages have an M2-like transcriptional profile and in this RCC cohort in which the transcriptional profiles of both subtypes aligned very closely (Supplementary Materials, Figure S3A–C). M0 macrophages are likely recruited to tumors by CAFs, which then promote their future polarization.

It has been demonstrated in prior work that the pro-angiogenic capacity of macrophages is determined in large part by biologically active MMP9 and low levels of TIMP1, which impairs the activity of MMP9. MMP9 is implicated as a driver of angiogenesis in ccRCC [37] and it is currently being investigated as a therapeutic target [38]. MMP9 is significantly differentially expressed by M0 macrophages based on LM22 signature gene expression analysis (Supplementary Materials Figure S1) [39] while IL4-induced M2 polarization reduces TIMP1. The combination of MMP9 produced by M0 macrophages and IL-4-induced M2 polarization reducing TIMP1 promotes angiogenesis in ccRCC and is a mechanistic explanation of the hallmark angiogenesis gene set being enriched in the M0-high cluster, which is also moderately enriched in M2-macrophages. Hypoxia was also noted to be a characteristic feature of the M0-enriched cluster in the gene set enrichment analysis. In addition to the dense fibrotic stroma and abnormal blood vessel architecture, M0-enrichment creates a hostile environment to PD-L1-expressing CD8+ effector T-lymphocytes, ultimately leading to decreased PD-L1 expression and poorer prognosis in this cohort. Indeed, M0 macrophages are associated with poor prognosis in ccRCC, with a greater abundance in higher-risk patients [40].

Our analysis is limited by its cross-sectional design, reflecting when the tumor was resected and sequenced. As a result, the dynamic influences on macrophage polarization and other complex TIME interactions and variability over time were not captured. Our analysis was based on bulk transcriptome RNA sequencing data and thus may not capture cellular level heterogeneity within the TME. Additionally, we now know that tumor architecture is important in defining function and biology, and this is well studied in kidney cancer, with CD8+ T cell location defining exhaustion state and the epithelial to mesenchymal transcriptional program co-localizing with macrophages at the tumor–normal interface, for example [41]. Importantly, we did not comprehensively address or examine all of the mechanisms contributing to resistance to ICB therapy, and we are limited by those mechanisms explored by the TIDE tool. Furthermore, we did not validate our findings in an ICB-treated cohort. We hope to address mechanisms of resistance to ICB more comprehensively in future work as we obtain more sequencing data from treatment-naive localized disease patients managed with ICB. Analysis by our group (Supplementary Materials, Figures S4 and S5) demonstrates that the immune microenvironment changes as patients develop metastatic disease and receive prior lines of therapy. For example, M0-macropgage content was found to dramatically decrease in those treated with systemic chemotherapy. A more appropriate ICB-treated population in which to validate our findings is those with localized disease treated with adjuvant immunotherapy, and such analysis can be performed as these data become more readily available. Notably, another limitation of using TIDE is that it was validated in melanoma and non-small-cell lung cancer. We do not have ground truth immunotherapy response data to validate TIDE’s findings; thus, we treated TIDE as hypothesis-generating in this study. Future studies using single-cell RNAseq (scRNASeq) and spatial transcriptomic techniques may provide further insights into the biology of ccRCC at higher resolution [42]. Nevertheless, this study lays the foundation for cell populations of interest and pathways to interrogate with higher fidelity in future studies to understand the underlying mechanisms of the TIME in kidney cancer biology as it pertains to optimizing systemic therapy regimens in patients at high risk of recurrence. For example, myeloid inflammatory subgroups have been previously shown to benefit from combination therapy with anti-VEGF and anti-PDL1 antibodies as opposed to checkpoint inhibition alone [43].

## 5. Conclusions

Our analysis revealed that M0 macrophages play a distinct role in early tumorigenesis of a more aggressive phenotype of kidney cancer, and further investigation of their role in RCC biology and exploration as a potential therapeutic target is warranted. The current paper has facilitated the generation of hypotheses and informs ongoing work including using organoids, single-cell and spatial analyses as well as drug testing to understand kidney cancer biology and discover novel therapeutic targets.

## Figures and Tables

**Table 1:** Demographic, pathologic, and molecular characteristics of non-metastatic patients in the TCGA-KIRC cohort, organized by the TIME cluster.

	Cluster 1 (*n* = 77)	Cluster 2 (*n* = 116)	Cluster 3 (*n* = 94)	Cluster 4 (*n* = 25)	Cluster 5 (*n* = 70)
Median Age (years)	60.9	61.8	59.4	62.4	60.2
Gender (M/F, %)	62/38	59/41	72/28	72/28	61/39
Race (W/B/A/NA, %)	96/4/0/0	95/3/1/2	93/3/2/2	96/0/4/0	96/1/3/0
Laterality (R/L, %)	61/39	53/47	53/47	60/40	46/54
Mutation Count (Min, Median, Max)	1, 48, 553	1, 48, 708	1, 48, 409	1, 48, 89	1, 48, 93
Fraction Genome Altered (Mean %)	15.9	13.3	16.1	23.4	15.9
Lab Parameters (↑/↓/WNL/NA %)					
Serum Calcium	3/44/23/30	1/41/27/32	0/49/17/34	0/60/16/24	1/43/26/30
Hemoglobin	1/52/32/14	2/46/39/14	1/45/33/21	0/56/36/8	0/56/39/6
Platelet Count	5/10/68/17	8/12/66/15	3/6/69/21	0/20/68/12	6/9/77/9
White Blood Cell Count	34/0/48/0	38/3/44/16	40/1/36/22	32/4/52/12	37/1/54/8
Lymph Nodes + (%)	0	8	16	10	23
Grade (%)					
G1	5	3	0	4	1
G2	57	46	54	32	37
G3	25	41	39	52	46
G4	13	9	5	12	16
TNM Stage Group (%)					
I	60	53	68	40	49
II	12	11	11	12	16
III	27	35	19	48	34
IV	1	0	1	0	1
Pathologic T Stage (%)					
T1, T1a, T1b	6, 26, 27	4, 32, 18	7, 38, 22	0, 20, 24	0, 20, 29
T2, T2a, T2b	10, 1, 0	7, 3, 1	11, 0, 0	12, 0, 0	14, 1, 1
T3, T3a, T3b, T3c	0, 17, 10, 0	1, 24, 8, 2	1, 17, 2, 0	0, 16, 28, 0	0, 21, 11, 0
T4	1	0	1	0	1
Mutations (% WT, MUT, NA)					
*TP53*	90/1/9	90/4/6	87/2/11	88/0/12	90/0/10
*VHL*	44/48/9	41/53/6	35/54/11	52/36/12	44/45/10
*PBRM1*	56/35/9	62/32/6	59/31/11	52/36/12	74.16.10
*SETD2*	75/16/9	87/7/6	82/7/11	76/12/12	84/6/10
*TCEB1*	91/0/9	93/1/6	88/1/11	88/0/12	89/1/10
Predicted ICB Response (%)	27	23	20	4	34

Abbreviations: ↑: value greater than normal range, ↓: value less than normal range, M: male, F: female. W: White, B: Black, A: Asian, NA: not available, R: right, L: left, WNL: within normal limits, WT: wild type, and MUT: mutant.

**Table 2:** Demographic, pathologic, and molecular characteristics of localized ccRCC patients in the CPTAC cohort.

	Cluster 1 (*n* = 9)	Cluster 2 (*n* = 9)	Cluster 3 (*n* = 25)	Cluster 4 (*n* = 17)	Cluster 5 (*n* = 28)	Cluster 6 (*n* = 11)
Median Age (years)	61.1	64.1	58.6	64.2	57.6	63.7
Gender (M/F, %)	78/22	78/22	84/16	65/35	64/36	73/27
Race (W/B/A/NA, %)	22/0/0/78	67/0/0/33	48/0/0/52	47/0/0/53	54/4/4 38	64/0/0/36
BMI (Mean)	25.3	28.3	33.2	32.4	31.9	30.8
Tumor Site (LP, M, UP, OTH, %)	11, 22, 33, 33	0, 11, 33, 56	12, 20, 32, 36	6, 35, 24, 35	14, 29, 29, 29	36, 18, 27, 18
Tumor Size (Mean, cm)	6.04	8.56	6.33	6.77	5.64	5.79
Grade (%)						
G1	0	0	0	24	7	0
G2	89	22	52	35	57	45
G3	11	44	44	41	29	55
G4	0	33	4	0	7	0
Pathologic Stage (%)						
I	44	33	40	53	50	55
II	11	11	16	18	7	18
III	22	22	40	24	32	27
IV	11	33	4	6	11	0
Pathologic T Stage (%)						
T1, T1a, T1b	0, 22, 22	0, 11, 22	0, 20, 20	0, 18, 35	0, 39, 14	0, 36, 27
T2, T2a, T2b	0, 11, 0	0, 11, 11	0, 8, 8	0, 18, 0, 0	0, 4, 4	0, 9, 9,
T3, T3a, T3b, T3c	11, 11, 0, 0	11, 11, 22, 0	4, 36, 4, 0	0, 18, 6, 0	4, 36, 0, 0	0, 18, 0
T4	11	0	0	6	0	0
Lab Parameters (↑/↓/WNL/NA %)						
Serum Calcium	0/0/11/89	0/0/56/44	0/8/16/76	0/12/18/70	4/4/18/75	0/18/0/82
Hemoglobin (HgB)	0/11/56/33	0/22/56/22	0/28/36/36	0/39/22/35	0/32/32/36	9/27/27/36
Platelets (PLT)	0/0/67/33	0/0/78/22		0/0/59/41	0/0/61/39	
White Blood Cells (WBC)	0/0/67/33	0/0/78/22	0/0/64/36	6/0/59/35	11/4/50/35	0/9/55/36
Laterality (L/R, %)	78/22	56/44	40/60	29/71	46/54	73/27
Margins Involved (%)	22	22	4	0	0	9
Residual Tumor						
R0	44	67	52	59	43	36
R1	0	0	4	0	0	0
R2	0	0	0	0	0	0
Rx	56	33	44	41	57	64
Pack Years (Mean)	22.9	18.3	34	36.5	26.3	14.7

Abbreviations: ↑: value above normal range, ↓: value less than normal range, M: male, F: female, BMI: body mass index, LP: lower pole, UP: upper pole, MID: middle, OTH: other, W: White, B: Black, A: Asian, NA: not available, R: right, L: left, WNL: within normal limits, WT: wild type, and MUT: mutant.

## Data Availability

All genomic and clinical data were obtained from publicly available sources. One can visit https://portal.gdc.cancer.gov/ and https://proteomic.datacommons.cancer.gov/pdc/browse to download the raw genomic and clinical data from the TCGA and CPTAC projects, respectively. Initially accessed on 12 February 2020.

## Supplementary Materials

The following supporting information can be downloaded at: https://www.mdpi.com/article/10.3390/cancers15235530/s1. Figure S1. Significantly differentially expressed genes from LM22 signature unique to M0 macrophages identified by comparing gene expression by each cell subset with the remaining subsets (e.g., Expression of gene X by M0 macrophages compared to all other cell subsets). Genes significantly expressed are denoted by number 1 and red color of cells. Figure S2. Heatmap displaying the relative abundance of immune cell subsets in the CPTAC dataset by cluster. The columns display the relative fraction of each individual immune cell, while the rows display unique identifiers within each cluster. Abbreviations: CD8: CD8 effector T cells, CD4: CD4 memory resting cells, MONO: monocytes, M0–M2: macrophage subsets. Figure S3. (A–C). Dot plots demonstrating the expression of various hallmark genes related to angiogenesis, cell cycle progression and epithelial-to-mesenchymal transition by various macrophage subsets (panel A. M0; panel B. M1; and panel C. M2) in the TCGA cohort. Given that these were gene sets of interest overexpressed by the M0 macrophage cluster in the primary analysis, we decided to see how expression of these gene sets compares across macrophage subsets. On the *x*-axis are genes of interest and on the *y*-axis is the log expression of the genes. Figure S4. Heatmap displaying the relative abundance of immune cell subsets in the CheckMate 010/025 and ImMotion 150 datasets. The columns display the relative fraction of each individual immune cell, while the rows display unique identifiers within each cluster. This analysis was subdivided by prior treatment patients received, including prior surgery, systemic therapy or if they were treatment naïve. Notable changes in the immune microenvironment based on prior therapy include lack of M0-macrophage enrichment in those receiving prior systemic therapy. Abbreviations: M0: M0 macrophages, M2: M2 macrophages, CD4-MR: CD4 memory resting cells. Figure S5. Dot plot displaying M0-macrophage content in patients based on prior therapy, with each dot representing a particular tumor specimen, fraction of M0 macrophages in the tumor specimen on the *y*-axis and prior-treatment on the *x*-axis. Again, notice the marked depletion of M0-macropgages in those patients who had undergone prior systemic therapy.
