# Supplementary material for: Analysis of the Tumor Immune Microenvironment (TIME) in Clear Cell Renal Cell Carcinoma (ccRCC) Reveals an M0 Macrophage-Enriched Subtype: An Exploration of Prognostic and Biological Characteristics of This Immune Phenotype
Source: Cancers (Basel). 2023 Nov 22;15(23):5530. doi: 10.3390/cancers15235530 (PMC10705373; doi:10.3390/cancers15235530)
Supplement: Supplementary file 1 [file cancers-15-05530-s001.zip › cancers-2473203-supplementary.pdf]

GENE	M0	M1	M2
ACP5	1	0	0
BHLHE41	1	0	0
C5AR1	1	0	0
CCDC102B	1	0	0
CCL22	1	0	0
CCL7	1	0	0
COL8A2	1	0	0
CSF1	1	0	0
CXCL3	1	0	0
CXCL5	1	0	0
CYP27A1	1	0	0
DCSTAMP	1	0	0
GPC4	1	0	0
HK3	1	0	0
IGSF6	1	0	0
MARCO	1	0	0
MMP9	1	0	0
NCF2	1	0	0
PLA2G7	1	0	0
PPBP	1	0	0
QPCT	1	0	0
SLAMF8	1	0	0
SLC12A8	1	0	0
TNFSF14	1	0	0
VNN1	1	0	0

Figure S1. Significantly differentially expressed genes from LM22 signature unique to M0 macrophages identified by comparing gene expression by each cell subset with the remaining subsets (e.g., Expression of gene X by M0 macrophages compared to all other cell subsets). Genes significantly expressed are denoted by number 1 and red color of cells.

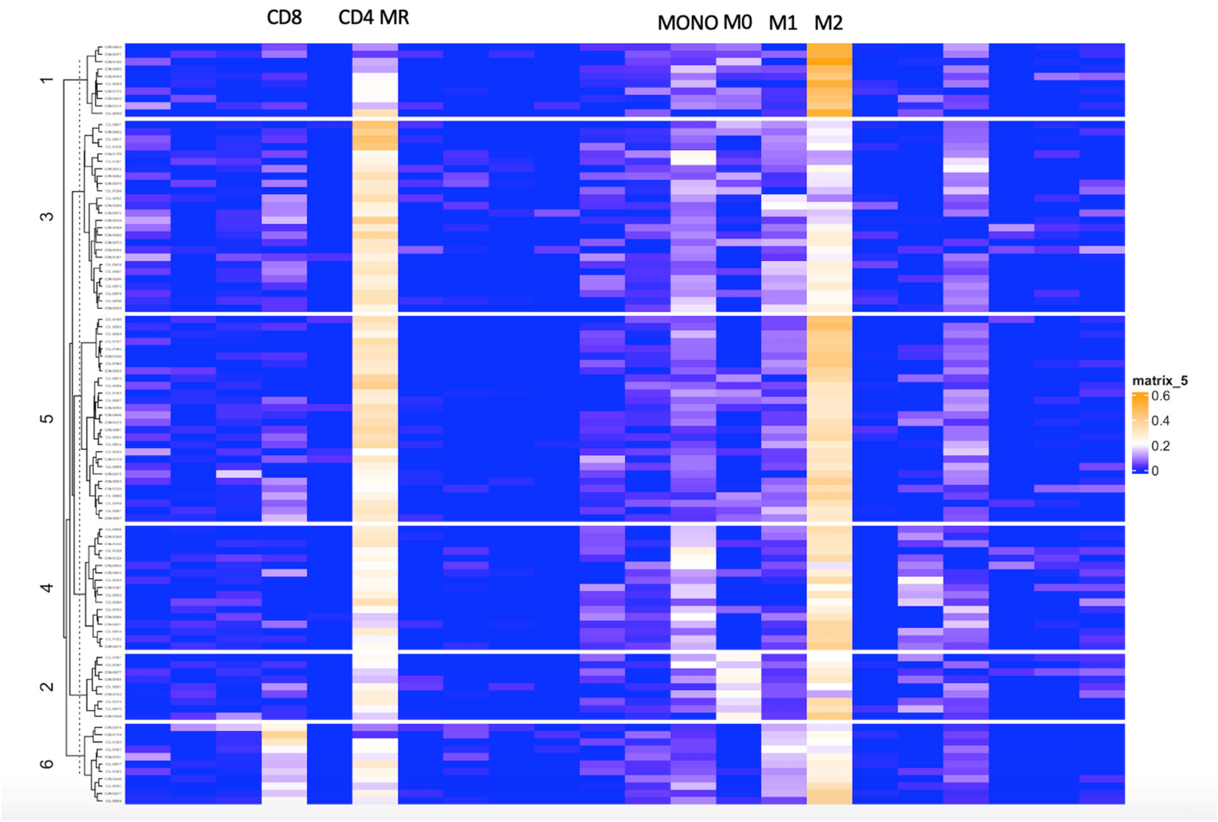

Figure S2. Heatmap displaying the relative abundance of immune cell subsets in the CPTAC dataset by cluster. The columns display the relative fraction of each individual immune cell, while the rows display unique identifiers within each cluster. **Abbreviations:** CD8: CD8 effector T cells, CD4: CD4 memory resting cells, MONO: monocytes, M0-M2: macrophage subsets.

A

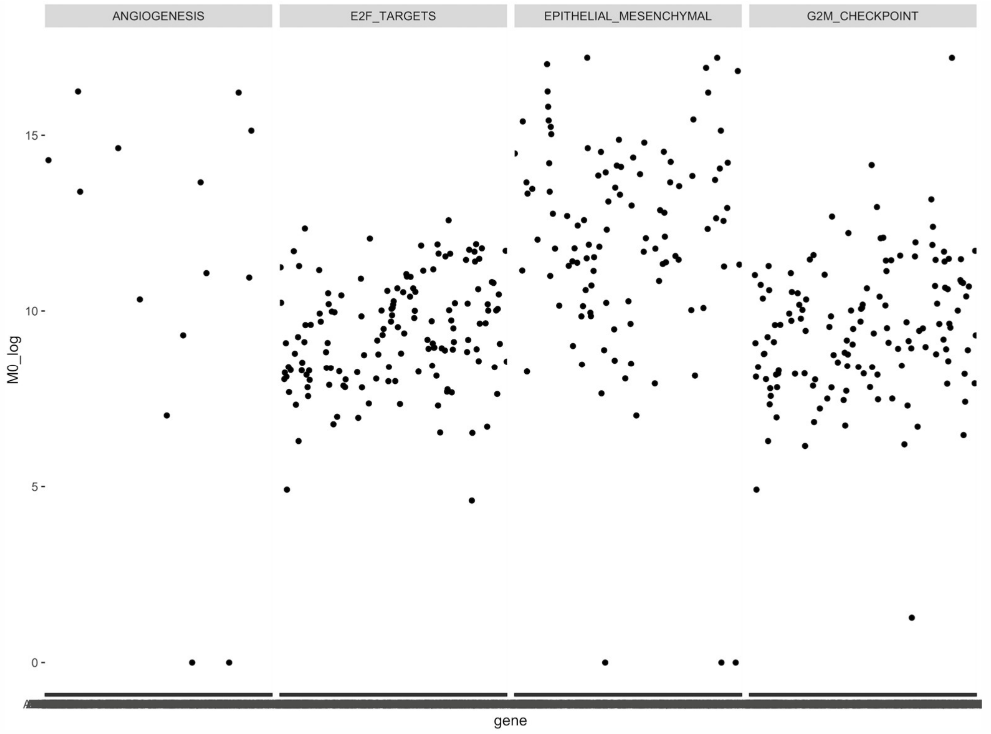
ANGIOGENESIS
E2F_TARGETS
EPITHELIAL_MESENCHYMAL
G2M_CHECKPOINT
M0_log
15
10
5
0
gene

B

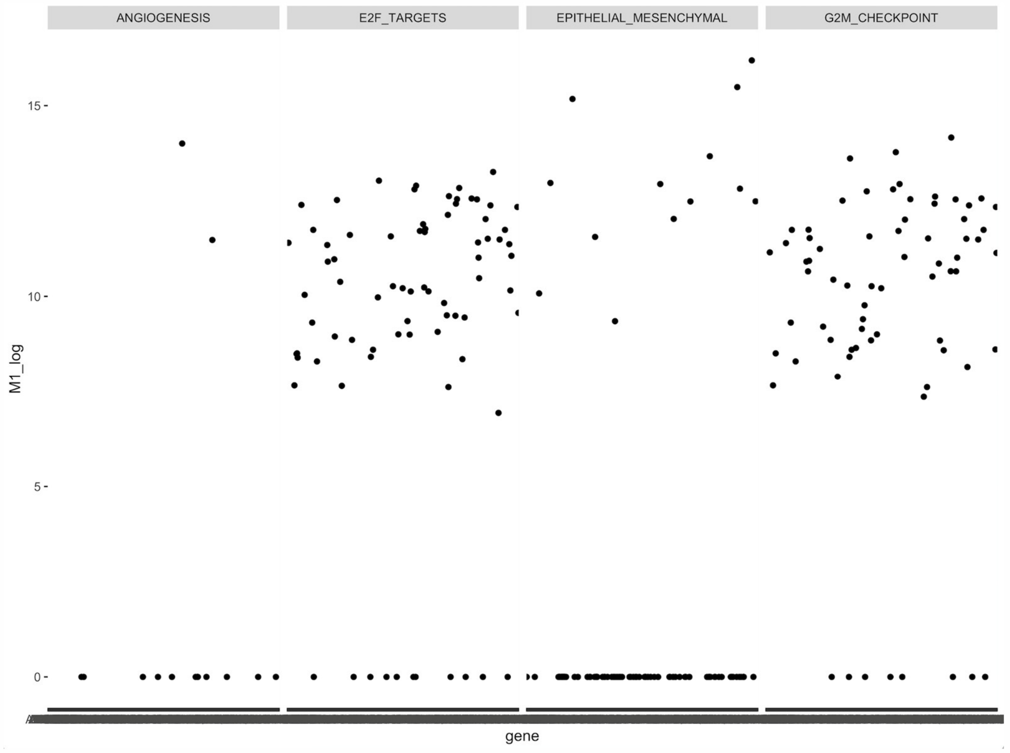
ANGIOGENESIS
E2F_TARGETS
EPITHELIAL_MESENCHYMAL
G2M_CHECKPOINT
M1_log
15
10
5
0
gene

C

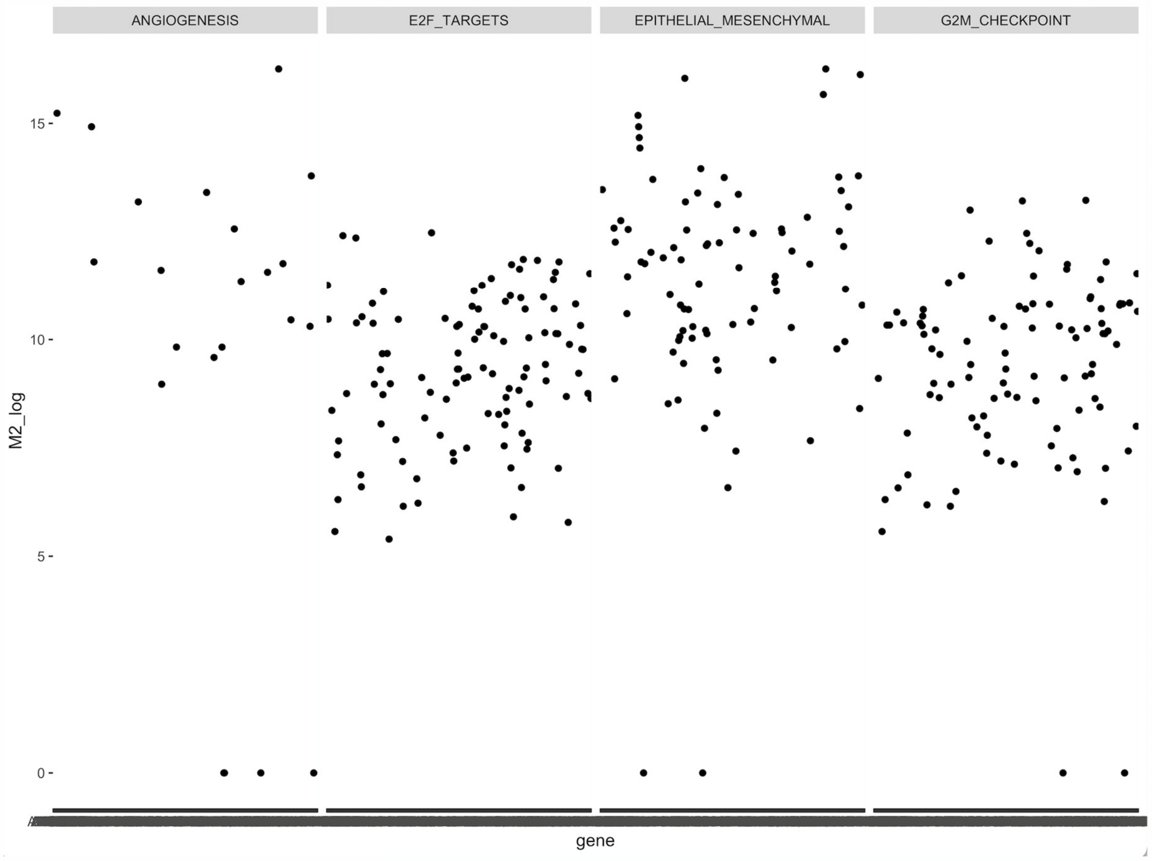

Figure S3 (a-c). Dot plots demonstrating the expression of various hallmark genes related to angiogenesis, cell cycle progression and epithelial-to-mesenchymal transition by various macrophage subsets (panel A. M0; panel B. M1; and panel C. M2) in the TCGA cohort. Given that these were gene sets of interest overexpressed by the M0 macrophage cluster in the primary analysis, we decided to see how expression of these gene sets compares across macrophage subsets. On the x-axis are genes of interest and on the y-axis is the log expression of the genes.

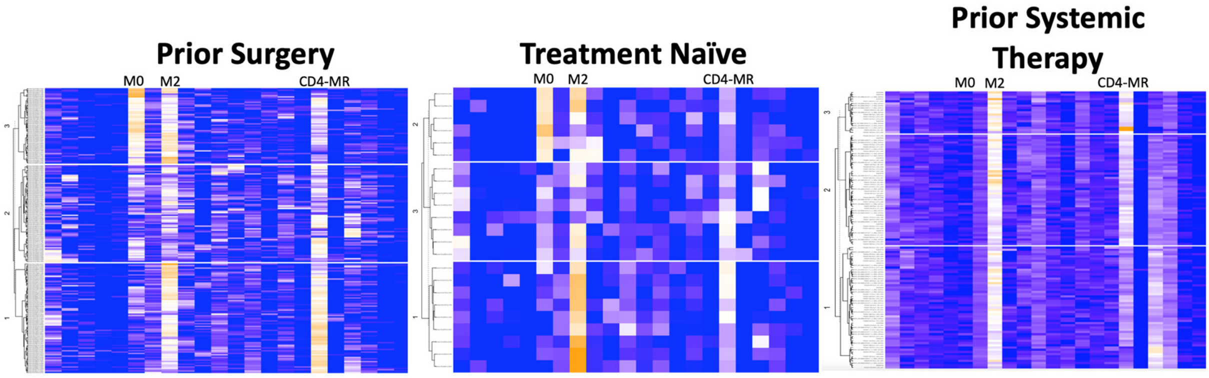

Figure S4. Heatmap displaying the relative abundance of immune cell subsets in the CheckMate 010/025 and ImMotion 150 datasets. The columns display the relative fraction of each individual immune cell, while the rows display unique identifiers within each cluster. This analysis was subdivided by prior treatment patients received, including prior surgery, systemic therapy or if they were treatment naïve. Notable changes in the immune microenvironment based on prior therapy include lack of M0-macrophage enrichment in those receiving prior systemic therapy. **Abbreviations:** M0: M0 macrophages, M2: M2 macrophages, CD4-MR: CD4 memory resting cells.

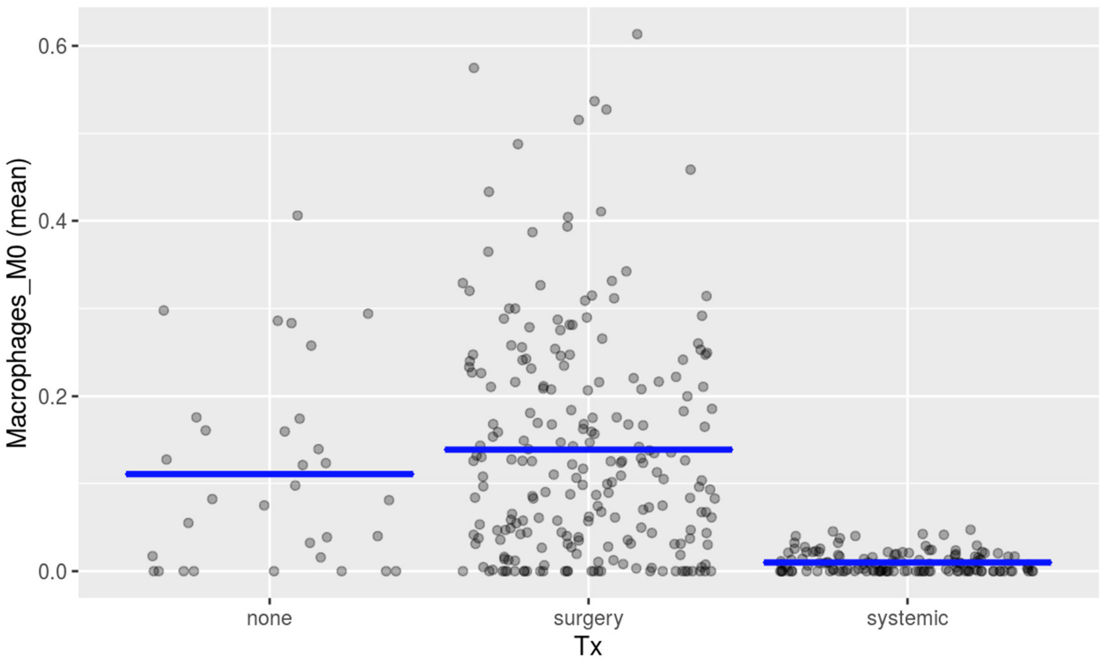

Figure S5. Dot plot displaying M0-macrophage content in patients based on prior therapy, with each dot representing a particular tumor specimen, fraction of M0 macrophages in the tumor specimen on the Y-axis and prior-treatment on the X-axis. Again, notice the marked depletion of M0-macropgages in those patients who had undergone prior systemic therapy.
